# From both sides of the needle: Understanding effective interventions for facilitating non-national immunization program vaccine decision making in China

**DOI:** 10.1080/21645515.2024.2389578

**Published:** 2024-08-22

**Authors:** Mingzhu Jiang, Xuanxuan Yan, Weixi Jiang, Haifeng Ma, Sijuan Zhou, Xiaohua Ying

**Affiliations:** School of Public Health, Fudan University, Shanghai, China

**Keywords:** Behaviour change wheel, China, in-depth interview, non-NIP vaccines, vaccination

## Abstract

Vaccination decisions are influenced by various psychological and practical factors. In China, non-National Immunization Program (non-NIP) vaccines, which are voluntary and self-paid, add uncertainty and autonomy to the decision-making process. Effective communication between providers and recipients is crucial but understudied. This study aims to integrate their perspectives, identify strategies for facilitating vaccination decisions, and analyze their mechanisms. From July to December 2023, semi-structured interviews were conducted with 17 caregivers and 12 vaccination providers across five Chinese provinces. Participants shared their experiences and decision-making processes regarding non-NIP vaccines. The Behaviour Change Wheel framework guided the analysis, utilizing iterative coding and directed content analysis. Thirteen Behavior Change Techniques were identified, with feedback, monitoring, and environmental restructuring being the most common. Key intervention functions included Persuasion, Education, and Training. We further mapped how these interventions influence non-NIP vaccine decisions. Capability was enhanced through education and effective communication, providing necessary knowledge and skills. Opportunity was increased via infrastructural improvements and societal support, making vaccines more accessible and endorsed by the community. Motivation was driven by clear communication of vaccination benefits and risks, reinforced by societal norms through public health messaging. By understanding the mechanisms influencing vaccination behaviors and interacting with stakeholders, tailored strategies can be developed. Healthcare providers can enhance service accessibility and offer evidence-based guidance with reminders, monitoring, and incentives to ensure compliance. For recipients, reliable information, sustained engagement, timely communication, and motivational opportunities are essential. A multi-dimensional approach involving multiple stakeholders is crucial for promoting non-NIP vaccine uptake.

## Background

Vaccination is the most cost-effective preventive intervention, particularly in controlling vaccine-preventable diseases (VPDs), reducing individual disease risks, and developing herd immunity both in stable times and during humanitarian crises.^1^ It directly impacts Sustainable Development Goals (SDG 3),supports 14 out of the 17 SDGs^2^ and contributes to achieving universal health coverage (UHC) by 2030.^3^ The focus on vaccine uptake has increased with the availability of more vaccines and combinations, alongside the rapid and widespread of ubiquitous global communication modes.Behavioral interventions, such as education campaigns, on-site vaccination, incentives, institutional recommendation, provider recommendation, reminder and recall, vaccine champion education campaigns, are often highlighted for their pivotal role in increasing vaccination rates and optimizing immunization effectiveness.^4,5^ It is crucial to explore how these interventions intersect with the complex web of interactions among stakeholders, which plays a significant role in shaping parents’ and informal caregivers’ decisions to vaccinate their children.

With a deeper understanding of the multidirectional nature of stakeholder interactions, attention is progressively shifting to focus on two key parties: the consumers or vaccine recipients, who are direct decision-makers, and the healthcare providers, who play a pivotal role as intermediaries, facilitating communication and trust between different levels of the healthcare system and the communities they serve. Research indicates that interaction between the vaccination recipients and providers is the keystone in fostering vaccine confidence and diminishing vaccine hesitancy.^6,7^ Quality immunization services also further require a strong focus on empowering and engaging both of them, they should be engaged in the planning, design, implementation and evaluation of these services.^8^ This involvement promotes structured, repeatable, and adaptable engagement, enabling informed decision-making and bridging gaps in understanding and service delivery.^9,10^ Additionally, their relationship is influenced by cultural and national contexts. Different countries have varying attitudes, requirements, and resources available for vaccination. Some countries may have rigorous vaccination mandates and substantial funding for immunization, while others might face challenges such as limited healthcare infrastructure, insufficient funding, or public skepticism about vaccines. Understanding these nuances is essential for developing effective interventions that resonate with diverse populations.

In China, non-NIP vaccines are typically optional and financially borne by the individual, characterized by a decision-making process fraught with inherent unpredictability and uncertainty.^11^ The factors influencing vaccination decisions are manifold and intricate, including access to healthcare or immunization services, deeply-rooted cultural beliefs, perception of disease severity, information, concerns, and confidence in vaccines.^12–16^ This decision-making scenario is sensitive to the socio-economic status of individuals, as the cost burden of non-NIP vaccines can be prohibitive for some. In most settings, the vaccine recipients are children under six years old, so the actual decision-makers are their parents and informal caregivers, who are now exposed to an increasingly wide and diverse range of information sources of mixed quality, posing new influences and challenges to their decision-making processes.^17^ The situation is further complicated by the rapid dissemination of information through digital and social media platforms, which often amplify uncertainties and create a more fragmented understanding of vaccines.

The existing research has concentrated on individual-level factors (e.g., affordability, knowledge)^18–21^ or has been limited to analyzing single provider recommendations.^22,23^ While these perspectives are indispensable, they do not fully capture the complex context and interactions among multiple stakeholders that influence vaccine coverage or the success of immunization programs. Research exploring a wide range of factors related to the healthcare environment in which caregivers or the public make decisions is needed, as well as requires a deep understanding of the interrelationships among different interventions to enhance their implementation and evaluation more effectively.

Currently, there exists a disparity in the availability of an all-encompassing methodology that systematically organizes and implements various interactive interventions, along with an understanding of the interconnections among different types of vaccination interventions. To facilitate the conceptual mapping of these interventions, a deep comprehension of their interrelations is essential. Behavior Science is well-suited to address this specific health behavior and multilevel factors and potential mechanisms associated with behavior change, and has been used in previous research examining facilitators or barriers to vaccination.^24–26^

In this study, we aim to align the perspectives of both providers and recipients to identify common strategies or interventions proven effective for promoting vaccination decisions and enhancing the uptake of non-NIP vaccines in China. By analyzing the pathways behind effective interventions, this research seeks to gain a deeper understanding of the multifaceted dynamic process of vaccination guided by theories of behavioral science, thereby providing more precise and targeted recommendations for public health practice.

## Methods

### Study design

From July to December 2023, we conducted semi-structured interviews with caregivers and vaccination service providers to gain in-depth insights into their experiences, perspectives, and decision-making processes about vaccination and provide a comprehensive understanding of the vaccination landscape from multiple viewpoints. The vaccination service provider refers to the qualified healthcare worker who provides vaccination services in the vaccination institution clinics within primary medical institutions, rather than general practitioners or specialist physicians. They are required to engage in vaccination work for three years or more and be familiar with the vaccination affairs within the institution. Given that the costs of non-NIP vaccines for children in China are mainly paid by parents, who have significant decision-making power, the interviewed caregiver refers to parents and needs to have experience in managing their children’s vaccinations.

### Study sites and recruitment

Taking into account economic indicators, regional characteristics, and geographical locations, we selected five provinces in the south (Guangdong), middle (Hubei), west (Sichuan and Yunnan), and northeast (Liaoning). Detailed information on these provinces is provided in Appendix 1. Within each selected province, we randomly chose one city and one county to reflect different administrative levels. Furthermore, within each county, we randomly selected one or two primary healthcare institutions to ensure a comprehensive sampling across varying contexts.

### Data collection

We developed an interview protocol that underwent assessment for suitability by our research team, complemented by expert consultation. Our questions included ones like: “What would you say are the key behaviors to promote the uptake of non-NIP vaccines? What is your understanding of vaccination? What was the experience like for you? What do you think influences your or other people’s decision to get vaccinated?.” The researchers invited 1 to 3 vaccination service providers to conduct the interviews within their respective health institutions. Concurrently, 1 to 3 caregivers were randomly selected for interviews at the vaccination clinics after their oral consent was obtained. No prior relationship was established before the study commenced.

We chose quiet, enclosed rooms for the interviews, ensuring that there was no one else present besides the participants and researchers. The interviews commenced with a brief introduction outlining the purpose of the research, adhered to the established protocol, and maintained a neutral and objective stance to mitigate any potential influence on the participants and minimize the interviewer effect. Each interview lasted approximately 45 to 60 minutes in Mandarin until thematic saturation was reached. The study received ethical approval from the Medical Ethics Committee of Fudan University.

Interviews were audio-recorded and transcribed verbatim without making field notes or returning them to participants for correction. To ensure confidentiality and anonymity, all data were stored in password-protected files accessible only to the research team. Personal identifiers were omitted from both audio and written transcripts, with numerical identifiers used during analysis (e.g., “1” for the first interview) to maintain participant privacy.

### Data analysis

**Figure 1.**
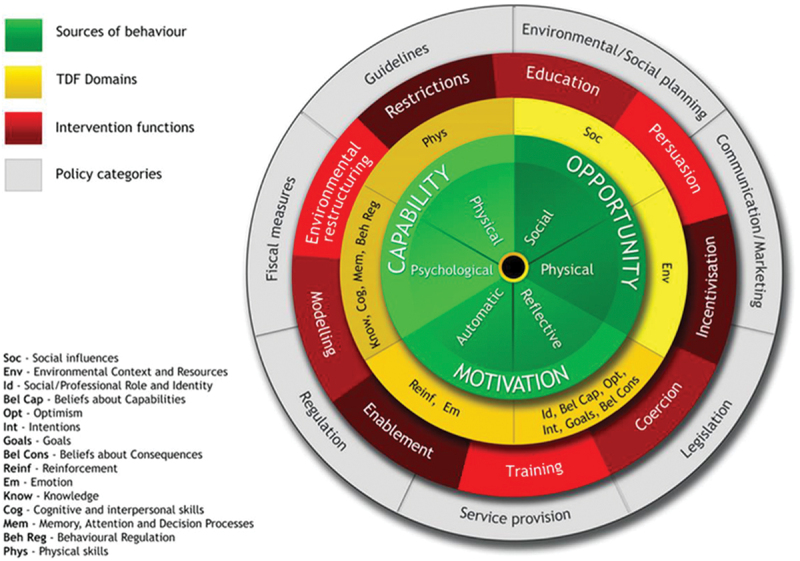
The behaviour change wheel (BCW) model.

Applying theory in the analysis of multi-component interventions is essential as it facilitates a thorough consideration of the factors influencing vaccination decisions and assists with understanding an intervention’s mechanisms of change.^27^ We utilized the Behaviour Change Wheel (BCW) to guide our analysis (Figure 1).

The Behavior Change Wheel (BCW) incorporates the Capability, Opportunity, Motivation, and Behavior (COM-B) model at its core, surrounded by nine intervention functions and seven policy categories, outlining a structured approach to understanding behaviors and determining appropriate intervention methods(the definition of intervention functions are shown in Appendix 2).^28^ Within this setup, the Behaviour Change Techniques (BCTs) are the fundamental, observable components crafted to modify behavioral regulatory processes. The standardized Behaviour Change Technique Taxonomy version 1 (BCTTv1) identifies 93 distinct BCTs, classified into 16 clusters that address diverse behavior change mechanisms, such as shaping knowledge, association, feedback, and monitoring^29^ (Appendix 3). These interconnected frameworks have been instrumental in identifying behavioral components relevant to vaccine uptake interventions and facilitating evidence synthesis.^30^

The two researchers (MZ and YX) first familiarized themselves with the materials and meticulously examined each transcript. They conducted an inductive analysis of the interview transcripts using NVivo 11 software (QSR International Pty Ltd, Victoria, Australia), applying directed content analysis^31^ and iterative methods. The originally released BCTTv1 served as our initial coding framework and themes were then identified and linked deductively to the COM-B model, culminating in the creation of a BCW logic model. Any inconsistencies were addressed and reconciled by a third researcher (XH) to ensure a unanimous agreement.

This article adheres to the adhered to the Consolidated Criteria for Reporting Qualitative Research (COREQ) checklist^32^ (Appendix 4).

## Results

### Participant characteristics

Finally, interviews were conducted with 29 individuals at six primary healthcare institutions across five cities in five provinces. This group comprised 17 female caregivers with an average monthly income of 5,611 yuan, most of whom have one child (*n* = 13) and hold educational qualifications below the undergraduate level (*n* = 10). Additionally, 12 healthcare workers participated, 7 of whom were female. Detailed demographic information is provided in Table 1.

**Table 1.** Basic information of interviews.

Type	Independent variable	N(%)
The caregivers	Sex	
	Female	17(100%)
	Male	0(0%)
	Reigion(Province)	
	Sichuan	4(24%)
	Yunnan	4(24%)
	Guandong	3(18%)
	Hubei	3(18%)
	Liaoning	3(18%)
	Occupational status	
	Employed	9(53%)
	Unemployed(including farming, homemaking, and other situations without a stable income)	8(47%)
	Education	
	Undergraduate	7(41%)
	Below undergraduate	10(59%)
The healthcare workers	Sex	
	Female	7(58%)
	Male	5(42%)
	Reigion (Province)	
	Sichuan	3(25%)
	Yunnan	3(25%)
	Guandong	2(17%)
	Hubei	2(17%)
	Liaoning	2(17%)

### Classification and description of BCTs

We labeled the 13 identified BCTs according to 9 specific categories within the BCT taxonomy (Table 2). Overall, the most commonly implemented BCTs included feedback and monitoring, along with restructuring the environment. Instruction, information provision, prompts, incentives, and other BCTs were utilized with the same frequency. Next, we will conduct detailed analysis of each BCT.

**Table 2.** Summary of BCTs identified in the vaccine interventions.

Intervention functions	BCT taxonomy	BCT label	Intervention strategies	Exemplar quote
Education Persuasion Training Enablement	2. Feedback and monitoring	2.2 Feedback on behavior	Combine traditional methods (in-person registration and phone reminders) with modern approaches (apps) to reduce the likelihood of missed vaccination appointments	After each vaccination, the institution’s worker informs us of the time for the next vaccination. Sometimes, if there is a long wait for the next vaccination, we can check the vaccination booklet or use an app. If an appointment is missed, a staff member from the institution will call to remind us. Thanks to these measures, my children have rarely missed a vaccination. *(Caregiver)*
Education Persuasion Training		2.3 Self-monitoring of behavior	Ensure individuals are reminded of and informed about their immunization schedules using paper vaccination records	In our institution, we have enhanced each vaccine recipient’s record book with detailed explanations of their immunization schedule. We use vivid colors and symbols such as stars and circles to highlight the availability of specific vaccines and the scheduling requirements for subsequent vaccinations. This method effectively informs them. Without this clear communication and marking, some parents would remain unaware of these vaccines. *(Healthcare worker)*
Education Persuasion Training		2.4 Feedback on outcome(s) of behavior	Inform patients of potential side effects and symptoms to monitor before receiving their vaccination, either verbally or via written educational materials, and ensure mandatory observation post-vaccination	Whenever a child is vaccinated today, we continue to educate parents to monitor their child’s temperature and check for any unusual reactions at the vaccination site. Parents often struggle to understand common side effects, and their concern for their child’s health is exceptionally high. Any new symptoms can cause significant anxiety, potentially affecting their decision to continue with vaccinations. We have encountered instances where parents, after experiencing undesirable effects from a previous vaccine, were reluctant to proceed with subsequent doses. *(Healthcare worker)*
Enablement	3. Social support	3.3 Social support (emotional)	Mitigate needle fear in clinical settings through the support of companions and positive encouragement	Some children and pregnant women are afraid of needles, so during early pregnancy education, we recommend that subsequent vaccinations be accompanied by family members. This approach serves two purposes: ensuring the safety of the vaccination and reducing their anxiety. *(Healthcare worker)*
Training Enablement	4. Shaping knowledge	4.1 Instruction on how to perform a behavior	Maintain the continuity and operability of vaccination services	After a newborn is delivered at a hospital, they are immediately vaccinated with the BCG and hepatitis B vaccines, and this information is entered into a connected system. The hospital updates the newborn’s information and informs the parents to visit a community health service center near their residence for subsequent vaccinations. Parental guidelines are provided to ensure a smooth transition from the hospital’s obstetric clinic to a regular vaccination clinic. *(Healthcare worker)*
Education Persuasion	5. Natural consequences	5.1 Information about health consequences	Provide information on vaccine-preventable diseases and the positive societal benefits of vaccination	We have discovered that vaccines are the most effective and cost-efficient means of preventing certain diseases. However, we find that communicating about the dangers of the diseases themselves is more effective than directly promoting the vaccines. This may be due to prevailing misunderstandings about non-national immunization program vaccines. *(Healthcare worker)*
Persuasion		5.2 Salience of consequences	Promote empathy and understanding through family experiences and community influences	We decided to vaccinate based on the observed protective effects of vaccines. When hand, foot, and mouth disease was prevalent in schools, our first child had been vaccinated and exhibited only mild symptoms during the outbreak. Consequently, we chose to give the same vaccine to our second child. *(Caregiver)*
Education	7. Associations	7.1 Prompts/cues	Offer a range of vaccination opportunities and information stimulus	After each vaccination, we write the vaccination schedule on the vaccine box and give it to the parents, allowing them to better understand the specific vaccine administered to their child. The box also includes an instruction manual, which might be more professional than our verbal explanations. Sometimes, while observing for half an hour post-vaccination, parents can read about the vaccines administered, the diseases they prevent, and other related information. *(Healthcare worker)* Additionally, our clinic is equipped with health education and disease prevention pamphlets, which are also available at the vaccination and observation areas. Parents can read these while waiting for the vaccination, enabling them to learn more about relevant health topics. *(Healthcare worker)*
Persuasion Enablement	9. Comparison of outcomes	9.1 Credible source	Acquire guidance, professional advice, and information provided by doctors	I believe that advice from doctors is crucial. For instance, we once had an elderly gentleman in our clinic who was advised by the doctor to get the PCV vaccine, and he immediately decided to go for it. When residents receive professional advice directly from doctors at the clinic, they tend to trust it more. In fact, doctors don’t necessarily need to recommend vaccines explicitly; merely mentioning available interventions, such as the flu vaccine, can significantly increase residents’ willingness to get vaccinated. *(Healthcare worker)*
Incentivisation	10. Reward and threat	10.8 Incentive (outcome)	Implement free vaccination of some vaccines (e.g. PCV, influenza, etc.)	At the time we implemented the free flu vaccination program, there was a particularly high number of people coming in for consultations. Our monitoring data also showed that the vaccination rate significantly increased that year. Moreover, this program greatly enhanced the public’s trust in our medical institution. We received a lot of positive feedback from community members, who expressed their gratitude for having access to the vaccine free of charge. *(Healthcare worker)*
Education Persuasion	11. Regulation	11.2 Reduce negative emotions	Engage with various information sources, enhance knowledge, and reduce concerns about vaccinations	We recognize that parents may have concerns about vaccinations and that family incomes vary. To address these issues, we offer prenatal classes for expectant mothers to equip them with early knowledge. Additionally, considering today’s parents often have high educational levels, many are proactive in seeking information. In fact, in numerous group chats, some individuals are even more informed than we are. *(Healthcare provider)* I use video platforms like TikTok to gather information, but as this can sometimes include a mix of reliable and unreliable sources, I follow several pediatricians. When their advice is consistent, I feel confident in trusting it rather than relying on a single opinion. Moreover, I have many expectant mothers around me, and we frequently share advice and recommendations on vaccinations. *(Caregiver)*
Environmental restructuring	12. Antecedents	12.1 Restructuring the physical environment	Establish digitalized vaccination clinics, improve vaccination environments, extend operational hours	It is now implementing process automation. After making an appointment, patients take a number and wait, then proceed to vaccination and subsequent observation. This process is highly efficient, and estimated waiting times can also be seen on the display screen. Moreover, our clinic has now opened vaccination services on weekends, offering parents more flexibility. Overall, the feedback has been quite positive. *(Healthcare provider)*
Environmental restructuring		12.2 Restructuring the social environment	Initiate collaborative campaigns and targeted outreach activities	Our vaccination efforts are progressing smoothly, primarily because the immunization staff have joined a general practitioner team, which regularly sends vaccine information to signed-up residents, emphasizing the integration of medical prevention. Additionally, we have leveraged the role of community workers, involving coordination with community centers and street offices. For key groups such as nursing homes, we provide information reminders. During specific seasons, we focus on promoting vaccines for diseases like chickenpox and hand-foot-mouth disease in schools, in conjunction with special vaccination events. *(Healthcare provider)*

The feedback and monitoring involve providing individuals with constructive feedback on their behavior and closely tracking their progress over time to reinforce positive behaviors or correct negative ones. According to interview results, these practices are most frequently used in promoting non-NIP vaccine uptake. The healthcare providers utilize three primary methods for reminders. The first is traditional phone reminders, where providers utilize digital information management platforms to conduct monthly analyses of vaccination statuses. By identifying individuals who have not been vaccinated on schedule, regular phone calls are made to inform them of missed vaccines and provide details regarding their next scheduled immunization. Although this function currently targets NIP vaccines, individuals can learn about other available vaccines, including those not covered by NIP during these interactions. This can increase awareness and motivate individuals to seek out additional vaccines. When healthcare providers engage with individuals about their vaccination schedules, it fosters greater awareness and education about vaccines in general. The process creates a framework and culture of vaccination that can naturally extend to include non-NIP vaccines, thus indirectly boosting their uptake.

The second method leverages modern information technology, primarily through mobile applications. Recipients are encouraged to download specific apps, such as Yue Miao and Xiao JinDou, which provide vaccination reminders and track vaccination records. In some regions, these software applications are promoted by the provincial Centers for Disease Control (CDC), while in others, they are provided by third-party IT companies.

In addition to digital tools, the third is paper or electronic vaccination certificates. In China, it is needed to keep timely records of each child’s vaccinations, including details such as the time of vaccination, type of vaccine, dosage, and manufacturer, ensuring the traceability of the information. Therefore, the health service providers also remind parents to bring vaccination certificates to facilitate better implementation of reminder functions. In one interviewed vaccination clinic, it innovatively places a conspicuous vaccination procedure chart on the first page of the vaccination certificate. This chart clearly outlines the vaccination process and marks essential details like vaccination times with prominent indicators, effectively engaging recipients’ attention toward their vaccination schedules. For parents, the vaccination record certificates act as an essential memorandum. They can review the log pages to remind themselves to timely take their children for vaccinations. Additionally, some applications facilitate the visualization of vaccine schedules for various age groups, enable the setting of reminders for forthcoming vaccination dates, and allow for the activation of alarms. These tools fulfill their roles in providing reminders and monitoring vaccination adherence.

Another frequently identified BCTs of restructuring the environment were related to modifying or reorganizing the physical or operational settings to enhance functionality and efficiency. In China, the implementation of standardized digital vaccination clinics exemplifies this approach. These clinics are designed to streamline the immunization process with a unified procedure, significantly reducing waiting times and improving the overall environment for immunization services. By utilizing digital tools, healthcare workers can manage appointments more effectively, ensuring a smoother and more efficient experience for all recipients. Furthermore, some clinics consider the vaccination demand, extend vaccination hours and optimize clinic schedules to increase the accessibility of vaccine services. For example, some clinics are now open on weekends to accommodate individuals who are unavailable during regular weekday hours. Specialized times are set for children’s vaccinations to stagger the flow of visitors and avoid overlap with adult vaccination. Additionally, staff rotations during lunch breaks are arranged to ensure that adequate vaccination resources are available at all times, maximizing the use of facilities and catering to the needs of the community. In addition to the aforementioned physical environment, a variety of effective measures have been implemented within the social environment. These involve community engagement and outreach efforts that have deepened interactions with key stakeholders, such as family doctors and community workers, who are pivotal in advancing community-driven projects. Furthermore, particular attention is paid to high-risk groups, such as students and the elderly. Exclusive vaccination and educational activities are conducted in schools and nursing homes, tailored to their specific settings, to ensure these vulnerable groups have access to vital vaccine information and services.

Instructions on how to perform certain behaviors were operationalized through resources offering comprehensive directives or information regarding the acquisition of non-NIP vaccines. In China, immediately after birth in medical institutions, newborns are required to receive the Bacillus Calmette – Guérin (BCG) vaccine and the hepatitis B (HepB) vaccine as their birth dose vaccine in life. Future vaccinations are then administered in vaccination clinics strategically distributed across primary healthcare facilities, including community health service centers and township health centers. As the initial contact for vaccination services, these medical institutions provide parents/caregivers with information about the nearest vaccination clinics to ensure the continuity of subsequent vaccination services. Concurrently, parents or caregivers have comprehensive access to a variety of resources, including links, tools, and detailed information that aid in determining the eligibility for various vaccine doses. These resources also support the processes of locating, scheduling, and accessing vaccinations, ensuring that caregivers are well-informed and prepared to navigate the complexities of vaccine administration for their children.

Social support was skillfully established by an interviewed vaccination clinic that facilitated the availability of nurses and support staff proficiently trained to alleviate the fear of needles. During mother classes, namely educational sessions aimed at early pregnancy, healthcare workers encouraged the presence of family members at future vaccinations, underscoring the vital role of familial unity in boosting vaccine uptake.

Certain incentives, specifically outcome-oriented, were implemented through free vaccination programs in economically developed regions. In these areas, targeted individuals could access specific vaccines at no cost. For instance, in Chengdu, free domestically produced bivalent HPV vaccines were provided to 13–14-year-old school girls, while Liaoning offered influenza vaccinations free of charge to registered elderly citizens over 60. Funded predominantly by local finances, these programs were bolstered by extensive promotional efforts and a high level of public awareness and acceptance of these vaccines, which in turn elicited a positive social reaction and significantly increased vaccination rates.

The BCT of prompts/cues describes elements designed to remind individuals to engage in specific behaviors, and are moderately employed across diverse strategies and resources. To ensure sufficient informational stimulation, several measures are in place. For instance, educational videos and informative brochures are shown in the observation area after vaccination, offering detailed insights into the importance of vaccination and associated health information to bolster visitors’ comprehension and awareness. Moreover, the essential details about non-NIP vaccines, such as costs, procedures, and potential side effects, are displayed at vaccination sites and clinics, guaranteeing that recipients are well-informed before vaccination. Additionally, the vaccine boxes and instructional manuals are distributed to parents, elucidating the ingredients, efficacy, and instructions for the use of the vaccines, thereby aiding parents in gaining a deeper understanding of the vaccines.

Other dispersed BCTs commonly reflect the transmission of information. These include information about health consequences, salience of consequences, credible source, and reduce negative emotions. Guidance or recommendations from doctors and healthcare providers are crucial, with physicians being particularly influential due to the heightened trust and respect parents have for their credibility and expertise compared to vaccination staff. In an age saturated with information, platforms like TikTok, and Wotobuy present content of varying quality, compelling consumers to sift through and validate information, ultimately gravitating toward the most reliable advice to guide their decisions. It is worth noticing that guidance from doctors or vaccination personnel about VPDs often carries more weight than mere details about the vaccines themselves. This effect is partly due to the perception among some parents in China that recommendations for non-NIP vaccines are profit-driven, potentially fostering skepticism and resistance. Moreover, educational campaigns that emphasize the severity of diseases and the efficacy of preventative measures tend to improve the acceptance of vaccinations. Personal experiences or those of acquaintances have an impact, particularly in families with multiple children. The experience of the first child or parents themselves, whether having contracted the related disease or having benefited from previous vaccinations, significantly heightens the probability of parents choosing non-NIP vaccines for their subsequent children. Particularly during the peak seasons for diseases such as influenza and hand, foot, and mouth disease, parents often engage in herd or spontaneous behaviors, leading to a significant increase in vaccination rates during these times. This highlights the profound impact of personal narratives and collective experiences in shaping healthcare decisions.

### BCT function and its interaction

Further, we identified Persuasion(*n* = 7), Education(*n* = 6), Training(*n* = 4), Enablement(*n* = 4), Environmental restructuring(*n* = 2), and Incentivisation (*n* = 1) as important components of interventions aimed at improving non-NIP vaccine uptake (Table 2). We incorporate this into the COM-B model, and from the viewpoints of both the vaccine recipients and the service providers, we elucidate how it fosters changes in non-NIP vaccine uptake behaviors.

When considering capability, the individuals or concerned must possess the requisite physical strength, knowledge, skills, and stamina to perform the behavior, mainly concerning psychological and physical capability. For parents, the primary motivation lies in enhancing their understanding of vaccines and the diseases they prevent. The consensus among interviewed parents is that vaccination protects children from infectious diseases by stimulating the immune system to produce antibodies. They were generally able to mention common non-NIP vaccines, such as HPV, influenza, herpes zoster, pneumococcal conjugate vaccine, and Enterovirus A71 (EV71). Their skills in planning and organizing their child’s vaccination schedule have been improved through various stimulations, including vaccination booklets, apps, and reminders from health institutions. For service providers, their abilities to remind, communicate, and convey trust have also been enhanced, primarily due to the development of information platforms and the training they receive from both superiors and their institutions.

For behavior to occur, the opportunity must be present within a supportive physical and social environment, this includes being physically accessible, affordable, socially acceptable, and there must be ample time available. The physical environment is predominantly shaped and enhanced by the vaccination service providers. In China, vaccination facilities are widespread, and the parents surveyed reported relative convenience in accessing these centers. The vaccination process, scheduling, waiting times, and the environmental standards of the facilities are well-regulated, particularly with the adoption of information technology, which significantly enhances the efficiency of the vaccination services. Additionally, the convenience and appeal of vaccination services have been improved. To accommodate the demand for accessible vaccine services, some clinics already offer drop-in sessions or appointments outside of normal working hours, a profoundly humane consideration. Regarding the social environment, medical institutions stimulate different sources of information through brochures, videos, and displays in waiting and vaccination areas, leveraging community strength for focused education and advocacy in strategic locations and specific populations. For parents, actively seeking information and social support profoundly enriches their knowledge acquisition through various avenues, including guidance from healthcare professionals and social influences, norms, and values imparted by family, friends, and community discussions. The quality and depth of information provided are crucial, such as vaccine schedules, ingredients, potential risks associated with different vaccines, factors that activate the children’s immune systems, contraindications, and the VPDs.

**Figure 2.**
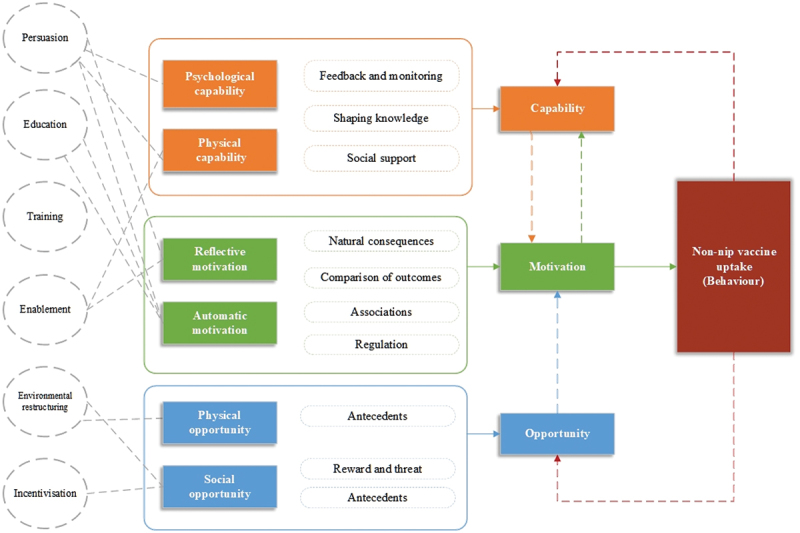
Logical model of thematic analysis mapped to the BCW in relation to COM-B, intervention functions, and BCTs for vaccination decision.

Finally, there must be a robust and compelling motivation driving individuals to non-NIP vaccine uptake. This means that individuals need to be significantly more motivated to engage in the act of getting vaccinated at the necessary moment than to abstain from it or to participate in any alternative activities that might compete for their attention or resources. Reflective motivation entails a process of conscious deliberation, where individuals actively consider the advantages and disadvantages of receiving a vaccine. They assess the perceived severity of the disease that the vaccine prevents, their vulnerability to contracting it, the efficacy of the vaccine in preventing illness, and any potential costs associated with getting vaccinated, such as time, discomfort, or financial expense. These factors collectively inform their decisions, shaping their intentions and plans regarding vaccination. Additionally, automatic motivation is driven by more instinctual and emotional factors that influence behavior on a subconscious level. This type of motivation includes the immediate and impulsive reactions to public health campaigns and messages, and the emotional comfort derived from aligning with the behavioral norms of one’s community. The figure below demonstrates how the COM integrates behavioral taxonomy and its functions, alongside an intervention mapping process stemming from it (Figure 2).

## Discussion

With an increasing global focus on vaccination accessibility and equity, China is proactively implementing the Immunization Agenda 2030 and Healthy China 2030, exploring the integration of vaccination with primary healthcare, catering to people-centered vaccine demands, ensuring extensive coverage and fairness, and promoting vaccinations across all stages of life while integrating them with essential health services.^33^ As previously mentioned, non-NIP vaccines in China are primarily self-funded and need voluntary decisions. In recent years, several noteworthy trends have emerged. The vast population and regional diversity create a high demand for a wide variety of vaccines, leading to a robust market for non-NIP vaccines. This demand has spurred continuous advancements in vaccine technology and development. Furthermore, the integration of digital platforms for health education and vaccination tracking has revolutionized how information is disseminated and how vaccines are administered. Social media, short videos, and other digital platforms have become powerful tools for raising awareness about the importance of vaccination, debunking myths, and encouraging voluntary participation in immunization programs.

As China endeavors to align its vaccination strategies with global initiatives and address new challenges, this study tries to connect policy initiatives to the tangible impacts on individuals receiving and providing vaccination services. We embark from two critical perspectives: the providers and the recipients of vaccination services, exploring and refining strategies to enhance the vaccination rates of non-NIP vaccines and their mechanisms of action. Applying a broad perspective from the start carries dual implications. It entails recognizing that both individual factors and contextual determinants shape vaccination behaviors, and it involves utilizing a comprehensive theoretical model to ensure that all Influence paths are considered, leaving no “blind spots” in the further analysis.

The results demonstrate that the synergistic engagement between service providers and recipients significantly enhances non-NIP vaccine uptake. This dynamic can be comprehended through the lens of push and pull. Push strategies are foundational in ensuring that the physical means and logistic supports for vaccination are robustly established and maintained. These efforts directly address supply-side elements influencing vaccination rates, including infrastructure development, service provider training, regulatory and scheduling flexibility, as well as systematic reminders and follow-ups. The pull measures are aimed at cultivating and intensifying demand for vaccinations by employing motivational, informational, and emotional appeals to individuals. This includes activities like enhanced educational outreach, engagement within communities and social networks, and leveraging emotional and cultural connections.

By synthesizing findings within the COM-B framework, we produced insights into the interplay of various factors that can significantly enhance vaccine uptake. Utilizing the COM-B model allowed us to delineate specific sub-components that influence each of the core elements – Capability, Opportunity, and Motivation – thereby offering a comprehensive understanding of their dynamic interactions. Research reveals that the promotion of non-NIP vaccine behaviors hinges crucially on the integrated effects of capability and motivation, which are individual determinants, and opportunity, which constitutes context determinants found in the physical and social environment. These elements are not isolated; rather, capability and opportunity synergistically influence motivation, which in turn, shapes behavior. This is consistent with previous studies, where substantial evidence has highlighted that knowledge and informational levels, social processes, and norms critically shape vaccination behaviors. Likewise, physical factors such as policies, institutional frameworks, and costs are also important determinants of vaccination behaviors, reinforcing the multifaceted nature of influences impacting vaccine uptake.^34^ The 13 interventions summarized here have demonstrated success in improving childhood Immunizations.^5^^,^^35–37^ Our study confirms that again that behavioral interventions can improve vaccine uptake.

As such, some interesting and progressive strategies are considered in planning efforts to maintain current immunization coverage among children and address existing gaps. One pivotal aspect that requires careful consideration is the application of digital tools. On one hand, these tools offer significant benefits by providing precise and up-to-date information about vaccine availability, safety, and efficacy, and by streamlining the processes for registration and scheduling appointments.^38^ Additionally, digital platforms can empower caregivers and healthcare workers, enabling them to express concerns, share experiences, and tap into support networks.^39^ However, the use of digital tools is not without its challenges. These platforms can also become conduits for the spread of misinformation, rumors, and stigmatization related to vaccines. Addressing these negative impacts is crucial for ensuring that digital tools serve as a positive force in vaccine decision-making processes.

Education and persuasion stand as the most commonly used functions of BCTs, yet their efficacy relies upon the quality and content of the information imparted. Before parents making determinations regarding vaccinations, the information should encompass not only the varieties of vaccines, their effectiveness, and potential adverse effects but also furnish detailed elucidations concerning specific diseases. This tailored education holds the potential to augment parents’ comprehension and assurance regarding the safeguarding capabilities of vaccines, empowering them to make enlightened decisions concerning vaccinations grounded in exhaustive and precise information.^22^

We have also observed that effective strategies are never implemented in isolation; they are always a combination of one or more intervention measures. We advocate for the utilization of a beneficial organizational framework for combined interventions, involving a combination of strategies that have different targets for improvement: caregiver motivation, accessibility, and services provided by providers and the healthcare system.

For service providers, it is essential to explore effective incentive mechanisms, which may include incorporating vaccination services into performance assessments, offering reasonable subsidies, providing training and certification, and awarding certificates of honor. The vaccination institutions should regularly offer relevant training programs that cover health education, assessment methods, and best practices for promoting non-NIP vaccine uptake.^40^ Furthermore, they keep in using innovative electronic information technologies, including real-time reminders to send personalized vaccination reminders and promptly provide feedback on vaccination status. By forming aggregated data nationwide, a truly national vaccination information system with cross-regional docking can be accessed.^41^ To effectively inform and engage the service recipients, strong community involvement and vaccine champions are valuable strategies. These efforts should be supported by communication campaigns and evidence-based interpersonal communication. Training healthcare providers, as well as community, faith, and industry leaders to serve as “vaccine champions” can enhance their ability to discuss the benefits of vaccination, address concerns, and counteract misinformation.^42^ To fully capitalize on the benefits of available vaccines, it is crucial to expand coverage to additional populations, including high-risk groups such as the elderly, individuals with chronic diseases, those in high-risk occupations, and adults overall. Moving forward, it is essential to prioritize initiatives aimed at educating the public and healthcare providers. This includes increasing awareness of adult vaccine utilization and conducting further operational research to thoroughly understand acceptance rates and vaccination practices among adults.^43–45^

This study had several limitations. Firstly, constrained by budgetary and time limitations, we utilized a sampling method to select five provinces for research. While the management of non-NIP vaccines in China generally exhibits consistency, innovative initiatives across different regions may not have been fully captured, as our study was confined to the sampled areas. Secondly, to respect privacy, only the gender of healthcare interviewees will be disclosed. Lastly, our mapping relied solely on the COM model, thereby lacking the analysis of mediating and transmission effects critical for understanding intervention effectiveness.

In future research, extending from our qualitative studies, there is a necessity to integrate both qualitative and quantitative methodologies. This can be facilitated through the use of surveys, scales, and other empirical tools to systematically evaluate the perspectives, perceptions, and assessments of vaccination strategies among vaccine recipients and healthcare providers. Moreover, investigating the tangible effects of vaccination strategies on vaccination rates and monitoring the dynamic variations in these rates across selected regions are crucial.

## Conclusion

The significance and efficacy of vaccination strategies are paramount in safeguarding public health against infectious diseases. Identifying the constituent elements of interventions and behaviors conducive to effective and efficient service provision is imperative, enabling thorough planning and execution. Our findings illuminate the efficacy of various strategies and delineate their propagation, underscoring the potential of behavioral interventions to promote vaccination decisions and non-NIP vaccine uptake within the Chinese context.

Regarding the involvement of healthcare providers, it is imperative to not only improve service accessibility but also establish a consistent, evidence-based approach to providing advice, complemented by ongoing reminders, monitoring, and incentives. For vaccine recipients, the reliability of information sources, consistent engagement, timely communication and opportunities for motivation are crucial.

Furthermore, there cannot be a one-size-fits-all strategy that enhances the uptake of non-NIP vaccines. Instead, a multifaceted strategy is required to effectively bolster vaccination decisions.

## Supplementary Material

Supplementary.docx

## Data Availability

All data and materials used are available from the corresponding author upon reasonable request.

## Abbreviations

BCG: Bacillus Calmette – Guérin
BCW: Behaviour Change Wheel
BCTs: the Behaviour Change Techniques
BCTTv1: the Behaviour Change Technique Taxonomy version 1
CDC: Centers for Disease Control
COM-B: Capability, Opportunity, Motivation, and Behavior
COREQ: the Consolidated Criteria for Reporting Qualitative Research
HPV: human papillomavirus vaccine
NIP: National Immunization Program
Non-NIP: Non-National Immunization Program
SDG: Sustainable Development Goals
UHC: universal health coverage
VPDs: vaccine-preventable diseases
