# Supplementary material for: From both sides of the needle: Understanding effective interventions for facilitating non-national immunization program vaccine decision making in China
Source: Hum Vaccin Immunother. 2024 Aug 22;20(1):2389578. doi: 10.1080/21645515.2024.2389578 (PMC11346547; doi:10.1080/21645515.2024.2389578)
Supplement: Supplementary.docx [file KHVI_A_2389578_SM8447.docx]

**Appendix 1: Basic information of sample sites**

| **Province** | **Population (in ten thousand)** | **Regional Gross Domestic Product**  **(in billion yuan)** | **Public budget revenue (in billion yuan)** | **Per capita disposable income**  **(in yuan)** | **Is there vaccine programs that funded by local fiscal ?** | **Programs Details** | **Vaccination service fee (per dose/yuan)** |
| --- | --- | --- | --- | --- | --- | --- | --- |
| Liaoning | 4197 | 28975.1 | 2525.07 | 36088.8 | Yes, not continuously ongoing | In 2021, Dalian city offered a free dose of influenza vaccine to all citywide residents aged 60 and above | 23 |
| Yunnan | 4693 | 28954.2 | 1949.46 | 26936.8 | Yes, partially ongoing | From 2018 to 2020, Kunming city offered free vaccination of the 23-valent pneumococcal vaccine for individuals aged 60 and above  From January 1, 2023 to December 31, 2025, Yuxi city provides free 2-valent HPV vaccination for female students in the first year of middle school | 15 |
| Sichuan | 8374 | 56749.8 | 4880.55 | 30679.2 | Yes, still ongoing | From 2021 to 2025, school girls aged 13-14 years old in Chengdu City can independently choose between domestic/imported bivalent and imported quadrivalent HPV vaccines, and are given a subsidy of 600 yuan per person | 20 |
| Guangdong | 12657 | 129118.6 | 13260.88 | 47064.6 | Yes, still ongoing | From September 2022, domestic 2-valent HPV vaccine will be administered to female students under the age of 14 who have Guangdong provincial school registration and have not received the HPV vaccine upon entering the first year of junior high school. | 21 |
| Hubei | 5844 | 53734.9 | 3281.13 | 32913.6 | No | / | 20 |

The above regional information is from the China Statistical Yearbook 2023, while the vaccine-related information is derived from interviews and official government websites.

**Appendix 2:**

**The definition of intervention functions in BCW**

| **Intervention function** | **Definition** |
| --- | --- |
| Education | Increasing knowledge or understanding |
| Persuasion | Using communication to induce positive or negative feelings or stimulate action |
| Incentivisation | Creating an expectation of reward |
| Coercion | Creating an expectation of punishment or cost |
| Training | Imparting skills |
| Restriction | Using rules to reduce the opportunity to engage in the target behaviour (or to increase the target behaviour by reducing the opportunity to engage in  competing behaviours) |
| Environmental restructuring | Changing the physical or social context |
| Modelling | Providing an example for people to aspire to or imitate |
| Enablement | Increasing means/reducing barriers to increase capability (beyond education and training) or opportunity (beyond environmental restructuring) |

**
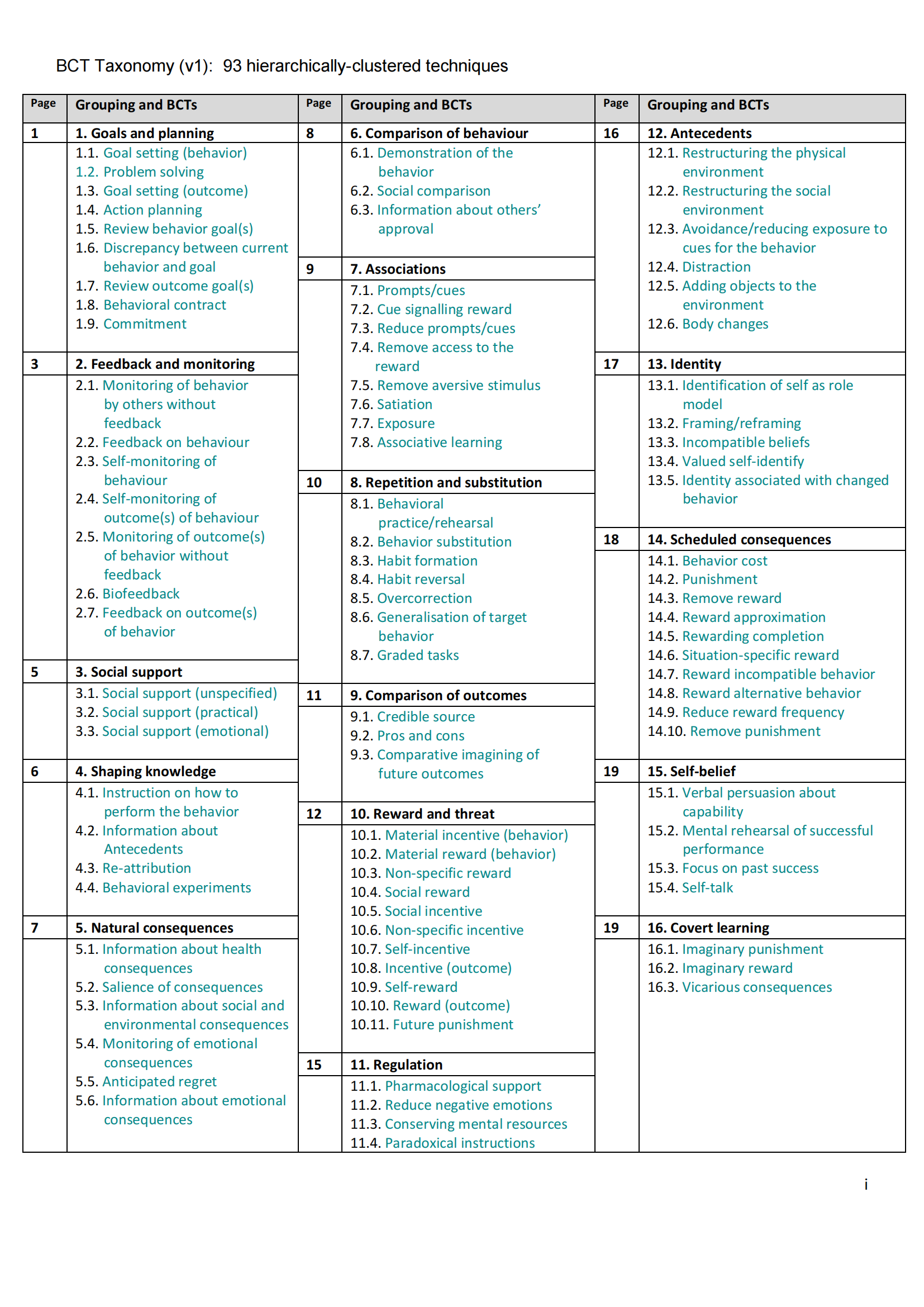
Appendix 3: The Standardized Behaviour Change Technique Taxonomy version 1 (BCTTv1)**

**Appendix 4: COREQ (Consolidated criteria for Reporting Qualitative research) Checklist**

| **Topic** | **Item No.** | **Guide Questions/Description** | **Reported on Page No.** |
| --- | --- | --- | --- |
| **Domain 1:Research team and reflexivity** | | | |
| *Personal characteristics* | | | |
| Interviewer/facilitator | 1 | Which author/s conducted the interview or focus group? | 2 |
| Credentials | 2 | What were the researcher’s credentials? E.g. PhD, MD | 2 |
| Occupation | 3 | What was their occupation at the time of the study? | 2 |
| Gender | 4 | Was the researcher male or female? | 4 |
| Experience and training | 5 | What experience or training did the researcher have? | 3 |
| *Relationship with participants* | | | |
| Relationship established | 6 | Was a relationship established prior to study commencement? | 3 |
| Participant knowledge of the interviewer | 7 | What did the participants know about the researcher?e.g. personal goals, reasons for doing the research | 3 |
| Interviewer characteristics | 8 | What characteristics were reported about the interviewer/facilitator? e.g. Bias, assumptions, reasons and interests in the research topic | 3 |
| **Domain 2:Study design** | | | |
| *Theoretical framework* | | | |
| Methodological orientation and Theory | 9 | What methodological orientation was stated to underpin the study? e.g. grounded theory, discourse analysis, ethnography, phenomenology,content analysis | 3 |
| Sampling | 10 | How were participants selected?e.g. purposive, convenience, consecutive, snowball | 2-3 |
| Method of approach | 11 | How were participants approached?e.g. face-to-face, telephone, mail, email | 3 |
| Sample size | 12 | How many participants were in the study? | 4 |
| Non-participation | 13 | How many people refused to participate or dropped out? Reasons? | 4 |
| *Setting* | | | |
| Setting of data collection | 14 | Where was the data collected? e.g. home, clinic, workplace | 3 |
| Presence of non-participants | 15 | Was anyone else present besides the participants and researchers? | 3 |
| Description of sample | 16 | What are the important characteristics of the sample? e.g. demographic data, date | 4 |
| *Data collection* | | | |
| Interview guide | 17 | Were questions, prompts, guides provided by the authors? Was it pilot tested? | 3 |
| Repeat interviews | 18 | Were repeat interviews carried out? If yes, how many? | 3 |
| Audio/visual recording | 19 | Did the research use audio or visual recording to collect the data? | 3 |
| Field notes | 20 | Were ﬁeld notes made during and/or after the interview or focus group? | 3 |
| Duration | 21 | What was the duration of the interviews or focus group? | 3 |
| Data saturation | 22 | Was data saturation discussed? | 4 |
| Transcripts returned | 23 | Were transcripts returned to participants for comment and/or correction? | 3 |
| **Domain 3:analysis and analysis** | | | |
| *Data analysis* | | | |
| Number of data coders | 24 | How many data coders coded the data? | 3 |
| Description of the coding tree | 25 | Did authors provide a description of the coding tree? | 3 |
| Derivation of themes | 26 | Were themes identiﬁed in advance or derived from the data? | 3 |
| Software | 27 | What software, if applicable, was used to manage the data? | 3 |
| Participant checking | 28 | Did participants provide feedback on the ﬁndings? |  |
| *Reporting* | | | |
| Quotations presented | 29 | Were participant quotations presented to illustrate the themes/ﬁndings? Was each quotation identiﬁed? e.g. participant number | 6-13 |
| Data and ﬁndings consistent | 30 | Was there consistency between the data presented and the ﬁndings? | 6-13 |
| Clarity of major themes | 31 | Were major themes clearly presented in the ﬁndings? | 6-13 |
| Clarity of minor themes | 32 | Is there a description of diverse cases or discussion of minor themes? | 6-13 |

Reference:

Tong, A., Sainsbury, P., & Craig, J. (2007). Consolidated criteria for reporting qualitative research (COREQ): a 32-item checklist for interviews and focus groups. International journal for quality in health care : journal of the International Society for Quality in Health Care, 19(6), 349–357.
